# Removal of Thiol-SAM on a Gold Surface for Re-Use of an Interdigitated Chain-Shaped Electrode

**DOI:** 10.3390/ma15062218

**Published:** 2022-03-17

**Authors:** Hien T. Ngoc Le, Le Minh Tu Phan, Sungbo Cho

**Affiliations:** 1 Department of Electronic Engineering, Gachon University, Seongnam-si 13120, Korea; ltnh1809@gachon.ac.kr; 2 School of Medicine and Pharmacy, The University of Danang, Danang 550000, Vietnam; plmtu@smp.udn.vn; 3 Department of Health Sciences and Technology, GAIHST, Gachon University, Incheon 21999, Korea

**Keywords:** interdigitated chain-shaped electrode, self-assembled monolayer, SAM removal, electrochemical impedance spectroscopy

## Abstract

The self-assembled monolayer (SAM) is the most common organic assembly utilized for the formation of the monolayers of alkane-thiolates on gold electrode, resulting in a wide range of applications for the modified SAM on gold in various research areas. This study examined the desorption of a SAM that was developed on the gold surface of an interdigitated chain-shaped electrode (the ICE, a unique electrode design, was fabricated by our group) with the goal of determining the most efficient strategy of SAM removal for the ICE to be re-used. A simple and proficient solution-based cleaning procedure was applied for the removal of a SAM on the gold surface of the ICE by using a sodium borohydride solution within short-term treatment, resulting in efficiency for the recovery of the originally electrochemical characteristic of ICE of 90.3%. The re-use of ICE after the removal process was confirmed by the successful re-deposition of a SAM onto the electrode surface, resulting in the high efficiency percentage of 90.1% for the reusability of ICE with the SAM modification. Electrochemical impedance spectroscopy (EIS) and cyclic voltammetry (CV) were used as tools to investigate the changes in the electrode interface at each stage of the SAM removal and the electrode recycling. X-ray photoelectron spectroscopy and Fourier-transform infrared spectroscopy were employed, being powerful spectrum techniques, for the characterization of the bonding structure and chemical state of the bare ICE and the modified ICE at each treatment step. Based on the comprehensive discussion of analytical chemistry from the obtained EIS and CV data in this study, we confirmed and proved the effectiveness of this promising method for the removal of a SAM from the ICE and the re-use of ICE in the field of material deposition, with the aims of saving money, improving experimental handling, and protecting the environment.

## 1. Introduction

Deposition processes are concerned with the deposition of materials, thin films, or composite coatings on a substrate. They have been applied for many applications in energy, biosensor, electronics, and semiconductor fields [1,2,3]. There are several deposition techniques, such as electrodeposition for the fabrication of Ni matrix micro- and nano-SiC composite coatings at room and elevated temperature [4], functionally graded Zn–Ni–Al_2_O_3_ coatings [5], and nickel–fullerene C60 composition coatings [6]; vacuum thermal evaporation [1,2]; electron beam evaporation [7]; laser beam evaporation [8] for depositing thin films; electrochemical deposition for developing thin layers, graphene, and nanoparticles [3,9]; and chemical-absorption for depositing a self-assembled monolayer on a gold substrate [10,11,12].

Molecular constituents from a solution adsorb onto a solid surface to form organic assemblies, which are known as self-assembled monolayers (SAMs) [13]. A SAM with a head group, a tail group, and a functional end group is representative of this. A wide range of self-assembling systems have been explored. Monolayers of alkane-thiolates on gold are presumably the most studied SAMs, since the –SH molecules of the head groups are tightly bound to the gold surface [10,11,12,13]. A SAM offers the most straightforward way to generate ultrathin, repeatable, oriented, and ordered monolayers that can preserve the activity of functionalized macro- or micro-molecules at the carboxylic acid (–COOH) terminal of the functional end group of the SAM [10,11]. Consequently, SAM has been used in a variety of research, including that of electrochemical biosensors [10,11,12], interface phenomena, biological and biochemical processes, electrochemistry, and molecular interactions [14,15,16,17]. As examples, gold electrode surfaces were modified with SAMs for binding different probe sequences to capture DNA target in order to establish a DNA biosensor for the diagnosis and treatment of an infectious disease [18]; a gold substrate was treated with a SAM by dip-coating and by patterning with a benchtop microdropper to explore neuronal adhesion through the precise and exclusive positioning of the neural cell bodies onto modified electrodes and inhibits, and at the same time, cellular adhesion in the surrounding insulator areas [19]; a gold surface was functionalized with the SAM to immobilize bacteriophages, in particular, for real-time monitoring of methicillin-resistant *Staphylococcus aureus* via surface plasmon resonance [20]; the immobilization of SAMs on gold fingers of an interdigitated chain-shaped electrode to develop various electrochemical biosensors for sensitive detection of protein biomarkers of Alzheimer’s disease [10,11,12].

The quartz crystal microbalance (QCM) is a traditionally technique to determine the adsorption of SAMs onto a gold surface. A model QCM device was used to detect the resonance frequency of the QCM chip in three stages: the bare chip, the chip immobilized with SAMs, and the chip after removing SAMs [21,22,23]. Recent QCM studies have reported: the formation of SAMs from stirred ferrocenylundecanethiol in hexane at room temperature, and the total frequency shift suggested SAMs formation [24]; the adsorption of two SAMs, i.e., octadecanethiol and octanethiol, in stirred hexane and cyclohexane solutions at room temperature over a broad range of concentrations, from 10^−3^ to 10^−1^ mM [25]; and the constitution of biotin-terminated thiol-SAMs on QCM for the construction of immunosensors [26]. Besides that, electrochemical impedance spectroscopy (EIS), a powerful electrochemical technique, has arisen and has been applied to characterizing the formation of the SAMs on gold electrode surfaces by monitoring the changes in the impedance at the electrode–electrolyte interface [10,11,12].

The typical rectangular interdigitated electrodes (IDEs) are constructed of two linear interdigitated electrodes with two connection tracks [27,28]. IDEs provide numerous benefits, such as working with low volumes of sample and avoiding the dreary polishing of solid electrodes [29]. These two configurations of IDEs enabled the wide utilization of IDEs in the characterization of changes in charge-transfer resistance and the double-layer capacitance at the electrode interface through the measurement of electrochemical impedance spectroscopy (EIS) [27]. However, a concentrated electric field at the edge is a side effect of IDEs, which reduces the signal quality during EIS measurements [10,30]. Particularly, in our previous study for monitoring the EIS of the cells on the IDE [31,32], it is well known that working area is not homogenous in the typical rectangular IDE, because the electric fields on the IDE are heterogeneous (the electric field is highly concentrated at the edge of the rectangular electrode). This means that the EIS measurements of the cell or material layers on the IDE are highly affected by the cells located at the electrode corners, resulting in reducing the EIS measurement sensitivity of the cell on the entire electrode area [31,32].

Therefore, in our recent studies [10,12], through correctly designing the electrode shape, by regulating the width or spacing of the electrode, the edge’s influence of electric field distribution has been avoided, leading to enhanced sensor area homogeneity on electrodes and precise EIS measurements of the electrode surface modifications. In previous studies [10,11,12], we fabricated an interdigitated chain-shaped electrode (ICE), a unique design. The interdigitated electrodes of ICE are designed in the shape of a chain containing numerous gold fingers of 5 µm to avoid the effect of the concentrated electric field at the edge. The distribution of electric fields in the areas between the ICE fingers is relatively uniform, as determined through using a COMSOL simulation [31], leading to enhanced homogeneity in the electrode working area, resulting in improved capabilities for electrode surface modification. Furthermore, the Warburg diffusion impedance in EIS has been completely eliminated by using ICE [10,11].

EIS has experienced a significant increase in popularity in recent years, owing to its exceptional sensitivity. The EIS technique is used to estimate the electrical properties of materials and their interfaces with electrodes that have been surface-modified [11,33]. This method has been extensively used in electrochemistry [34,35], biomedical applications [36,37], materials science [38], and other fields. EIS is a type of impedance measurement using alternating current polarography, which includes electrochemical reactions, and results in signals corresponding to the charge-transfer resistance and the double-layer capacitance at the electrode interface [39].

Our past research has demonstrated that a SAM is the main components used to successfully modify the gold surface of the ICE, which has been used in the construction of a variety of electrochemical biosensors in the field of sensing technology [10,11,12]. Besides that, the formation of a SAM on the gold surface has been utilized as a platform for the development of analytical tools in electronics, such as introducing a SAM as a counterbalancing dipole to the work function of the electrode in order to modify organic thin film transistors [40], using a SAM as a molecular junction between two electrodes with gold to shrink the size of electronics by replacing traditional thin film transistors with ones based on SAMs [41], modifying graphene with SAMs in the construction of graphene-based field effect transistors (GFETs) to reduce power consumption and use in very high frequency electronic devices [42,43,44], and patterning of SAMs on metal surfaces in applications of organic solar cells for modifying cathodes to block holes [45]. However, each ICE with a modified SAM and the electronic devices based on the platform SAM–gold were discarded after only one usage in each application, since it is hard to remove the SAM from the gold surface, leading to electrode waste, manufacturing costs, and an environmental impact. The development of technologies to regenerate the underlying gold will enable the electrode surface to be re-used, resulting in cost savings, environmental protection, and improved experimental handling. In an ideal situation, the SAM should be entirely eliminated from the surface without affecting the gold substrate.

To remove the SAM molecules from gold substrates, there are a variety of approaches available. Ozone and UV radiation are approaches that can be used in the presence of air to decompose the thiol compounds of the SAM [46]. However, the limitation of these procedures would be that the fluctuation must be spread evenly over the surface to be wiped, and portions that are shaded from the light may not have been efficiently wiped down. Heat treatment is a practical method for removing thiol molecules from air [47], but it still needs high temperatures (of over 200 °C) to be used for a reasonable timeframe, which could harm the gold substrate. SAMs have recently been shown to dissolve when exposed to a laser beam [48]. Due to the fact that it could only eliminate a tiny amount of a SAM at a time, this approach has limitations for real implementations.

It is highly useful to operate with liquids, since they allow many samples to be cleaned uniformly, while also reaching complex surfaces. Electrochemical cleaning is the most commonly used solution-based cleaning procedure. Whenever a negative voltage is given to the gold substrate, the SAM’s molecules begin to desorb, which is known as electrochemical desorption. It is common practice to immerse the SAM-coated gold substrate in an electrolyte solution with a pH that is basic or neutral [49]. Due to the fact that some alkane-thiolates are re-absorbed on the gold surface as a result of electrochemical cleaning, the potentials used and the time spent conducting the test are essential considerations [50].

In this study, we introduce a simple and efficient method for removing SAM from the gold surface of an ICE by using a sodium borohydride (NaBH_4_) solution. The ICE with the SAM modification was immersed in an NaBH_4_ solution for a short time to entirely remove the SAM on the ICE surface, and to recover the original electrochemical properties of the ICE. Furthermore, an applicable re-use of ICE after the SAM removal process was performed by the re-deposition of the SAM on the electrode surface. EIS and cyclic voltammetry were used to characterize the electrochemical behavior, and the changes in the electrode interface in the SAM removal stage and in the ICE re-use stage. The two powerful spectroscopy techniques of X-ray photoelectron spectroscopy and Fourier-transform infrared spectroscopy were utilized to characterize the electronic structure and chemical bonding of the bare ICE and the electrode modification at each stage. Additionally, the efficiencies of the removal of the SAM on the ICE surface and the re-use of ICE in the material deposition were calculated in this paper. We also propose an easy and new process for cleaning the bare ICE with NaBH_4_ solution, prior to beginning the electrode modifications, which is described in the following section.

## 2. Materials and Methods

### 2.1. Materials

6-Mercaptohexanoic acid (MHA, 90%), sodium borohydride (NaBH_4_, powder, ≥98.0%), and potassium ferrocyanide/ferricyanide ([Fe(CN)_6_]^3−/4−^, powder) were purchased from Sigma–Aldrich (Seoul, Korea). Potassium chloride (KCl, extra pure) was bought from Daejung Chemicals and Metals Co., Ltd., (Siheung, Korea). Phosphate-Buffered Saline (PBS, 1X, liquid, pH 7.4) was purchased from Welgene Inc. (Gyeongsan, Korea). De-ionized (DI) H_2_O was obtained from Water Purification System, PURESCIENCE (Seongnam, Korea). Ethanol (extra pure, 94%) was provided by OCI Company Ltd., (Seoul, Korea).

### 2.2. Manufacture of the Interdigitated Chain Shaped-Electrode (ICE)

**Figure 1:**
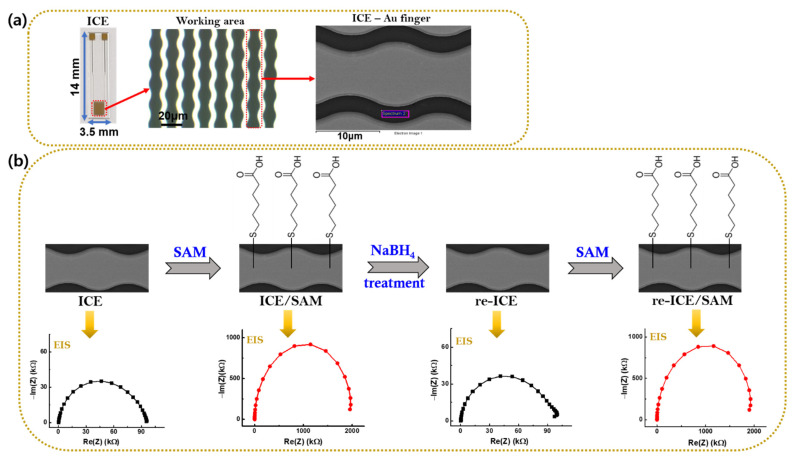
(**a**) Picture of ICE, a working area microscopic image of ICE containing several fingers, and a scanning electron microscopy (SEM) image of an Au finger of ICE. (**b**) Deposition of the SAM, removal of the SAM (by NaBH_4_), and re-deposition of the SAM procedures for the Au surface of ICE, respectively, and the corresponding EIS results at each stage.

The ICE was manufactured on a glass slide substrate of (14 mm × 3.5 mm). The electron beam evaporator system (SEE-5, ULTECH, Daegu, South Korea) was used to deposit a 25 nm thick adhesive Ti layer (deposition rate of 1 A s^−1^, emission current of 68–80 mA), and a 50 nm thick Au electrode layer (deposition rate of 1 A s^−1^, emission current of 55–74 mA). Next, the lift-off process was used to generate the paired electrode finger, including the space of 5 μm and the width of 5 μm, for the working and reference electrodes, respectively. Figure 1a shows a photo of ICE including the working and reference electrodes, and microscopic images of the ICE containing Au fingers.

### 2.3. Deposition of the Self-Assembled Monolayer (SAM) on the ICE. Removing the SAM on the Surface of ICE. Re-Depositing the SAM on the ICE

Before developing the SAM on the ICE surface, the bare ICE was cleaned with 0.5 M NaBH_4_ solution in 1:1 H_2_O/Ethanol and dried under nitrogen flow to discard impurities on the ICE surface.

Subsequently, the bare ICE was dipped in 100 mM MHA solution for 2 h at room temperature (RT; 25 °C) to create the SAM on the Au surface of ICE (ICE/SAM) through the bonding between gold (Au) of ICE and sulfur (S) at the head group of the SAM, as shown in Figure 1.

To re-use the ICE, the ICE/SAM was immersed in 0.5 M NaBH_4_ solution for 10 min at RT to remove the SAM on the ICE surface (re-ICE) and return the ICE to its original state.

To approve the re-use of ICE, the SAM was again deposited on the re-ICE (re-ICE/SAM) by dipping the re-ICE in a 100 mM MHA solution for 2 h at room temperature (25 °C), and then it was washed with extra pure ethanol and DI H_2_O. This procedure is similar to the deposition of the SAM on bare ICE, as described above, which is a quality control process for the successful formation of the SAM on the gold surface of ICE, as shown in the Results and Discussion section.

### 2.4. Structural Characterization

X-ray photoelectron spectroscopy (XPS, Thermo Scientific MultiLab 2000, East Grinstead, UK) with X-ray sources of a twin anode (Al K_α_, hν = 1486.6 eV) gun and a monochromatic gun was used to characterize the chemical state and the electronic structure of the bare electrode and the electrode modification.

Fourier-transform infrared spectroscopy (FT-IR, a wavenumber range of 400–4000 cm^−1^, scanning speed of 2 mm s^−1^, data interval of 0.964233 cm^−1^, resolution of 4 cm^−1^, Jasco-4600, Jasco Inc., Easton, MD, USA) was used to characterize the functional and bonding groups on the electrode surface. The FT-IR result was obtained by measuring three consecutive runs for each sample.

### 2.5. Electrochemical Characterization

BioLogic’s potentiostat/galvanostat instrument (SP-200, Seyssinet-Pariset, France) was used to investigate the electrochemical impedance spectroscopy (EIS) and the cyclic voltammetry (CV) measurements of the ICE and the ICE modification. EIS was performed in a two-electrode configuration with an input sinus amplitude of 50 mV and the applied frequency range of 1 MHz to 100 mHz, and CV was performed in a three-electrode configuration with an external Ag/AgCl electrode, in 1 mM K[Fe(CN)_6_]^3−/4−^ containing 0.1 M KCl.

## 3. Results and Discussion

**Figure 2:**
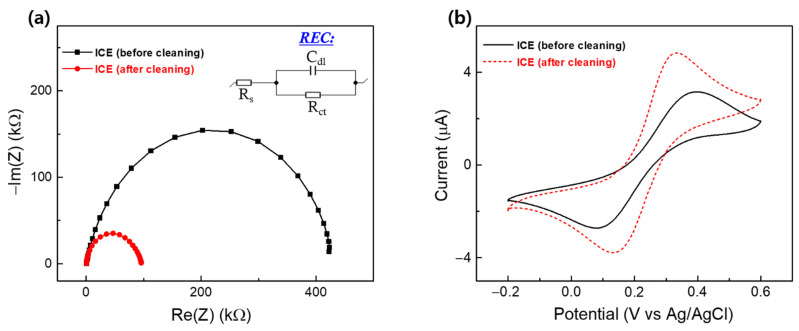
(**a**) EIS and (**b**) CV results of ICE, before and after the cleaning process.

Cleaning ICE is an important step to remove numerous contaminants on the electrode surface and enhance the quality of the active Au surface of ICE for the chemical modification. Therefore, the bare ICE was cleaned with 0.5 M NaBH_4_ solution to remove dust and impurities existing on the surface. Figure 2 shows the EIS and CV of the ICE before and after the cleaning process. EIS results express the changes in the interfacial electrode surface in the electrolyte of 1 mM K[Fe(CN)_6_]^3−/4−^ containing 0.1 M KCl in a Nyquist plot of the image impedance (−Im(Z)) vs. the real impedance (Re(Z)), as shown in Figure 2a. The meanings of Nyquist plots were described using the Randle’s equivalent circuit model (REC) (Figure 2a inset), which included three parameters: R_s_, corresponding to the solution resistance which can be found by reading the real axis (Re(Z)) value at the high frequency intercept; R_ct_, corresponding to the interfacial charge-transfer resistance which equals the semicircular diameter of the Nyquist plot; C_dl_, corresponding to the double-layer capacitance which is in parallel with the R_ct_.

Using the REC for fitting the measured Nyquist plots in Figure 2a, the obtained R_ct_ value of ICE decreased significantly to 86 kΩ after the cleaning step, as compared to the high R_ct_ value (390 kΩ) of ICE before the cleaning step. This decrease in R_ct_ was due to the easy transport of the K[Fe(CN)_6_]^3−/4−^ redox probes toward the electrode surface; this result indicated that the movement obstruction of redox probes was prevented by elimination of the impurities that existed on the electrode surface, demonstrating the successful removal of contaminants on the electrode surface, and that the original electrochemical behavior of the ICE had been brought back. CV of the ICE after cleaning (Figure 2b) showed the increase in current intensity, re-confirming the enhancement in the electrochemical properties of ICE. Recent studies of gold electrode cleaning procedures, such as electrochemical cleaning (via applying cyclic voltammetry between −1.0 and 1.3 V, scan rate of 0.1 V s^−1^ in a solution of 0.05 M H_2_SO_4_) [51,52,53,54] and heat treatment (via increasing temperature) [55], also displayed an increase in current intensity in the CV of gold electrode after the cleaning step, agreeing with the tendency of the obtained CV in this report, and proving the improvement in the quality of bare ICE after using the NaBH_4_ for cleaning.

### 3.1. XPS and FT-IR Results of ICE, ICE/SAM, and Re-ICE

**Figure 3:**
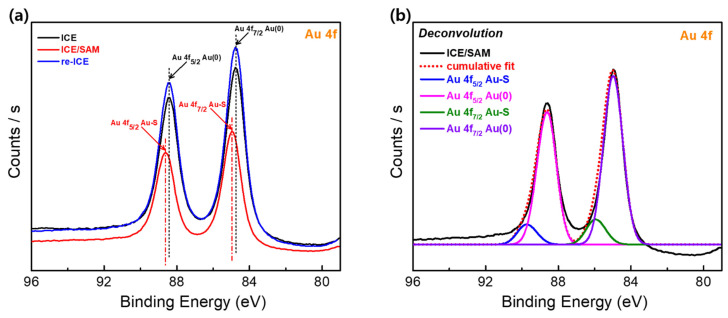
(**a**) XPS results of the ICE, ICE/SAM, and re-ICE. (**b**) Deconvolution of the Au 4f spectra of ICE/SAM.

To identify the change in the Au surface of ICE after the SAM modification and the SAM removal (as described in Section 2.3), the Au 4f spectra of the ICE, ICE/SAM, and re-ICE samples (Figure 3) were identified by using XPS, since XPS is a powerful technique to analyze the chemical state and electronic structure of an electrode surface [56]. As seen in Figure 3a, the Au 4f spectra of the bare ICE had the two typical peaks of bulk metallic gold Au(0), of the Au 4f_7/2_ Au(0) at 84 eV and the Au 4f_5/2_ Au(0) at 88 eV of binding energy, respectively [57]. After the deposition of the SAM, a shift in the Au 4f peaks was observed for the ICE/SAM sample (Figure 3a); this phenomenon is attributed to the formation of Au–S bonding between the Au surface of ICE and the terminal S group of the SAM. As seen in Figure 3b, the deconvolution of the Au 4f spectra of the ICE/SAM indicates two peaks of metallic gold of Au 4f_7/2_ Au(0) (84 eV) and Au 4f_5/2_ Au(0) (88 eV), along with the peaks that occurred for Au 4f_7/2_ Au–S (85 eV) and Au 4f_5/2_ Au–S (89 eV) [57], demonstrating the successful deposition of the SAM on the Au surface of the bare ICE. Subsequently, the Au 4f spectra of the ICE after the removal of the SAM from the electrode surface—the re-ICE sample (Figure 3a)—showed the disappearance of the Au–S peaks, and the return of the two peaks of Au(0) at 84 and 88 eV, as compared to the Au 4f spectra of the ICE and ICE/SAM, indicating that using the NaBH_4_ treatment brought back the ICE’s original characteristics.

**Figure 4:**
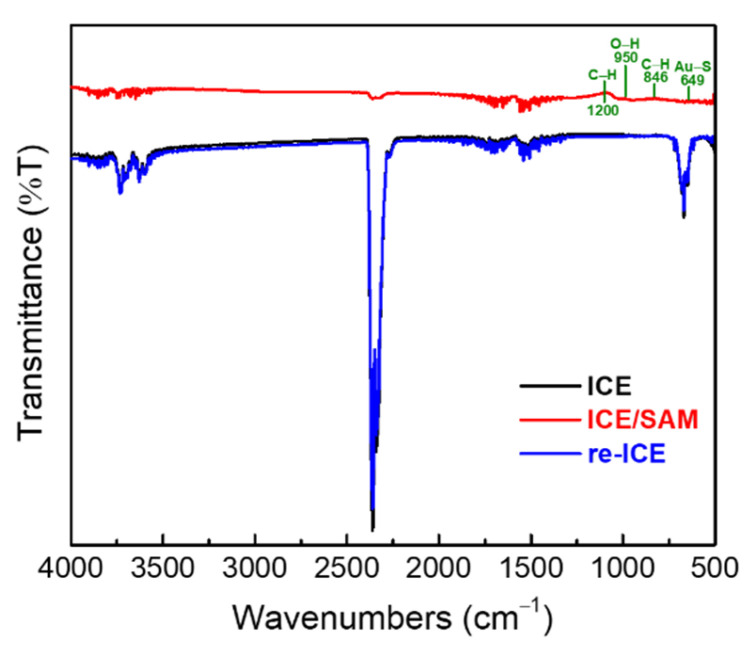
FT-IR results of the ICE, ICE/SAM, and re-ICE.

FT-IR was used to identify the bonding groups of the electrode and the modified electrode. Figure 4 shows the FT-IR spectra of the ICE, ICE/SAM, and re-ICE. The spectra of ICE/SAM show the formation of the covalent bonding of Au–S and the stretch mode of C–S groups of the SAM in the band range (500 to 850) cm^−1^, and the O–H deformation mode and methyl antisymmetric bending mode of the SAM in the band (950 to 1400) cm^−1^, respectively, demonstrating the deposition of the SAM on the Au surface of the bare ICE [58,59]. The re-ICE showed the FT-IR spectra that match the spectra of the bare ICE in all regions of wavenumbers after the SAM removal by NaBH_4_. This result indicated the successfully reorganization of the ICE after the SAM deposition and removal process, respectively.

### 3.2. Electrochemical Characterization of the ICE, ICE/SAM, and Re-ICE, and the Application of Re-ICE for the Deposition of the SAM on the Electrode Surface

**Figure 5:**
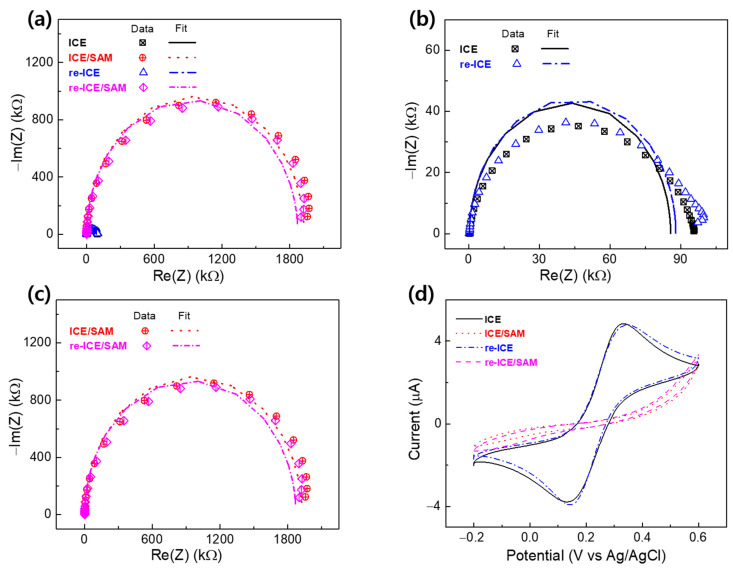
(**a**–**c**) EIS, expressed by Nyquist plots, and (**d**) the CV results of ICE, ICE/SAM, re-ICE, re-ICE/SAM in 1 mM K[Fe(CN)_6_]^3−/4−^ containing 0.1 M KCl. EIS spectra included the fitting data.

The EIS tests of ICE, ICE/SAM, re-ICE, and re-ICE/SAM were performed in 1 mM K[Fe(CN)_6_]^3−/4−^ containing 0.1 M KCl, and results are expressed through Nyquist plots, as shown in Figure 5a–c. Using REC to fit the EIS data, the values of the three parameters of R_ct_, C_dl_, and R_s_ were obtained, and are displayed in Table 1. The fitted R_ct_ value of the ICE was 86 kΩ. Significant enhancement in R_ct_ was observed after the addition of the SAM to the ICE surface (Figure 5a and Table 1), and the obtained R_ct_ value of ICE/SAM was 1900 kΩ. This increment in the R_ct_ was attributed to the non-conductivity of the long hydrocarbon chains and the carboxylic acids group of the SAM, which led to impeding the movement of the K[Fe(CN)_6_]^3−/4−^ redox couple to the ICE surface. As seen in Figure 5d, after SAM modification on the ICE, the CV result showed a rapid decrease in current intensity, indicating the reduced conductivity of the electrode due to the formation of the SAM on the electrode surface, agreeing with the EIS results.

Figure 5a,b shows the EIS results of the re-ICE after the removal of the SAM from the ICE surface with the treatment of NaBH_4_ solution. The Nyquist plot of the re-ICE demonstrates the expressive decrease in the R_ct_ with a value of 87 kΩ. This decrease in R_ct_ was due to the easy transportation of the K[Fe(CN)_6_]^3−/4−^ redox couple toward the electrode surface; the hindrance of the transfer of the redox probe to the surface of electrode was removed, which was caused by the elimination of the SAM on the ICE surface. It is observed that the R_ct_ value of the re-ICE (87 kΩ) approximately equals the R_ct_ of the bare ICE (86 kΩ), demonstrating that removal of the SAM from the ICE was successfully completed by the treatment with NaBH_4_ solution. The re-confirmation of the elimination of the SAM from the ICE’s surface was also performed by CV measurement. The CV result of the re-ICE after removing SAM in Figure 5d indicates a significant increase in the current intensity, and equals the current intensity of the CV result of the bare ICE, re-confirming the removal of the SAM from the ICE, and the restoration of the original electrochemical behaviors of ICE.

After the successful removal of the SAM from the ICE, the re-ICE was used to immobilize the SAM for the purpose of demonstrating the ICE’s reusability, as described in Section 2.3. Figure 5a,c shows that after the addition of the SAM to the re-ICE, the R_ct_ in the Nyquist plot of the re-ICE/SAM showed a remarkable increment (1870 kΩ in Table 1), as compared to the R_ct_ of the re-ICE (87 kΩ) and the ICE (86 kΩ). This increment in R_ct_ confirmed the formation of the SAM insulator on the surface of re-ICE, leading to delaying the transfer of the K[Fe(CN)_6_]^3−/4−^ redox couple to the electrode surface. The re-ICE/SAM showed the approximate R_ct_ value, as compared to the R_ct_ of the ICE/SAM, as seen in Figure 5c and Table 1, indicating the reusability of the ICE in the field of material deposition. The CV result of the re-ICE/SAM in Figure 5d displays the rapid decline of current intensity compared to the CV of the re-ICE and shows a similar tendency to the CV result of the ICE/SAM, which was related to the non-conductivity of the SAM EIS results. All EIS and CV results indicate or confirm the successful restoration of the original properties, and the reusability of ICE in material deposition.

### 3.3. Efficiency Percentage

To evaluate the possible factors for the recovery of ICE’s characteristic after the removal of the SAM and the re-use of ICE with the SAM modification, the efficiency percentage was determined by calculation, as described in the following evaluation:

The efficiency percentage of the ICE recovery after SAM removal can be calculated as follows:

Efficiency (%) = (R_ICE_/R_re-ICE_) × 100, (1)

where R_ICE_ is the R_ct_ of the bare ICE, and R_re-ICE_ is the R_ct_ of the re-ICE after the removal of the SAM.

The efficiency percentage of the reusability of ICE with the SAM modification on the surface can be calculated as follows:

Efficiency (%) = (R_ICE/SAM_/R_re-ICE/SAM_) × 100 (2)

where R_ICE/SAM_ is the R_ct_ of the ICE/SAM with the addition of the SAM to the ICE, and R_re-ICE/SAM_ is the R_ct_ of the re-ICE/SAM with the re-modification of re-ICE with a SAM.

**Figure 6:**
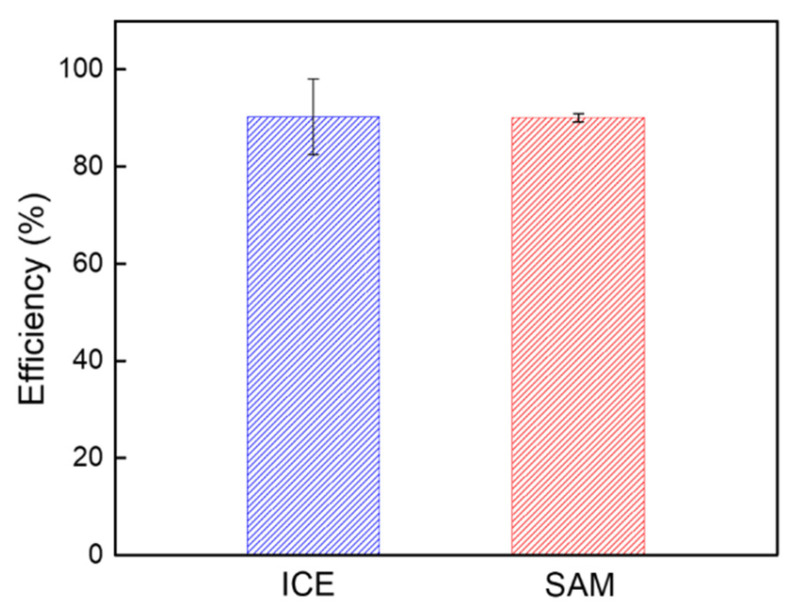
Efficiency percentage (%) for the re-use ICE and the re-deposition SAM. Columns and bars represent the averages and standard deviations of the data (*n* = 3).

Therefore, as shown in Figure 6, the efficiency percentage of the recovery of the ICE was found to be 90.3%, and the efficiency percentage of the recovery of the SAM modification was determined to be 90.1%, demonstrating the high efficiency results of the restoration of the ICE and the reusability of ICE for the SAM modification. These results confirmed the effectiveness of the method for the removal of thiol SAM on the gold surface of ICE, which was explored in this study, allowing us an estimation of the efficacy of this means for the property rebuilding of the ICE for the material deposition applications.

## 4. Conclusions

The aim of this work was to study the most effective method for removing the modified SAM on an ICE’s gold surface in order to determine the plausibility of a reuse procedure for the electrode.

Therefore, in this study, a convenient, simple, and efficiency strategy to remove SAM on the gold surface of ICE was explored by treatment for 10 min with a solution of NaBH_4_ with the purpose of reusing ICE to save money, protect the environment, and expeditiously manage experiments.

The full removal of the SAM and the good reusability of ICE with re-addition of the SAM were evaluated and confirmed by analyzing the electrochemical measurements, such as EIS and CV, and by using microscopy techniques such as XPS and FT-IR.

The high-efficiency percentages for the recovery of the electrochemical characteristics of ICE, and for the re-deposition of the SAM on the ICE surface, were also determined, and found to be 90.3% and 90.1%, respectively, indicating a well-founded approach for the regeneration of ICE’s gold surface in applications of material deposition, providing advantages in terms of cost savings, experimental control, and environmental protection.

A new and concise route for cleaning the bare electrode surface by applying a NaBH_4_ solution was also presented in this paper.

## Figures and Tables

**Table 1:** EIS parameters of the electrode and the modified electrode, extrapolated by fitting the measured spectra in Figure 5 to the REC.

Electrode	R_ct_ (kΩ)	C_dl_ (F)	R_s_ (kΩ)
ICE	86 ± 1.0	(11.8 × 10^−7^) ± 10^−5^	0.36 ± 10^−4^
ICE/SAM	1900 ± 0.1	(0.53 × 10^−7^) ± 10^−3^	0.35 ± 10^−5^
re-ICE	87 ± 2.0	(11.7 × 10^−7^) ± 10^−5^	0.35 ± 10^−4^
re-ICE/SAM	1870 ± 0.1	(0.54 × 10^−7^) ± 10^−3^	0.35 ± 10^−4^

## Data Availability

Not applicable.
